# Two-Generation Toxicity Study of the Antioxidant Compound Propyl-Propane Thiosulfonate (PTSO)

**DOI:** 10.3390/antiox13030350

**Published:** 2024-03-15

**Authors:** Antonio Cascajosa-Lira, Remedios Guzmán-Guillén, Silvia Pichardo, Alberto Baños, Jose M. de la Torre, Nahum Ayala-Soldado, M. Rosario Moyano-Salvago, Isabel Ortiz-Jaraba, Ana M. Cameán, Angeles Jos

**Affiliations:** 1 Área de Toxicología, Facultad de Farmacia, Universidad de Sevilla, 41012 Sevilla, Spain; aclira@us.es (A.C.-L.); rguzman1@us.es (R.G.-G.); camean@us.es (A.M.C.); angelesjos@us.es (A.J.); 2 DMC Research Center, Camino de Jayena, 82, 18620 Granada, Spain; abarjona@domca.com (A.B.); josemanuel.delatorre@dmcrc.com (J.M.d.l.T.); 3 Departamento de Anatomía y Anatomía Patológica Comparadas y Toxicología, UIC Zoonosis y Enfermedades Emergentes ENZOEM, Facultad de Veterinaria, Universidad de Córdoba, Campus de Rabanales, Edificio Darwin, 14071 Córdoba, Spain; nahum.ayala@uco.es (N.A.-S.); r.moyano@uco.es (M.R.M.-S.); 4 Departamento de Medicina y Cirugía Animal, Facultad de Veterinaria, Universidad de Córdoba, Campus de Rabanales, Edificio Darwin, 14071 Córdoba, Spain; v52orjai@uco.es

**Keywords:** two-generation toxicity, natural additive, antioxidant, organosulfur compound, safety assessment, in vivo, *Allium* sp., thiosulfonate

## Abstract

Propyl-propane thiosulfonate (PTSO), an antioxidant organosulfur compound present in the genus *Allium*, has become a potential natural additive for food and feed, as well as a possible biopesticide for pest control in plants. A toxicological assessment is necessary to verify its safety for livestock, consumers, and the environment. As part of the risk assessment of PTSO, this study was designed to explore its potential reproductive toxicity in mice following the OECD 416 guideline. The investigation spans two generations to comprehensively evaluate potential reproductive, teratogenic, and hereditary effects. A total of 80 CD1 mice per sex and generation were subjected to PTSO exposure during three phases (premating, gestation, and lactation). This evaluation encompassed three dose levels: 14, 28, and 55 mg PTSO/kg b.w./day, administered through the feed. No clinical changes or mortality attributed to the administration of PTSO were observed in the study. Some changes in the body weight and food consumption were observed, but not related to sex or in a dose-dependent manner. The two parental generations (F0, F1) exhibited normal reproductive performance, and the offspring (F1 and F2) were born without any abnormalities. The serum sexual hormone levels (progesterone -P-, testosterone -T-, estradiol -E2-, follicular stimulating hormone -FSH-, and luteinizing hormone -LH-) were in a normal range. Although significant changes were observed in the sperm analysis in the case of F0 group, no variation was found for F1 group, and no alterations in fertility were recorded either. The absolute organ weights and relative organ weight/body weight and organ weight/brain weight ratios, and the complete histopathological study, showed no significant alterations in males and females for all the generations considered. Considering all the results obtained, PTSO is not considered a reproductive or developmental toxicant in mice under the assayed conditions. These results support the good safety profile of PTSO for its potential application in the agrifood sector.

## 1. Introduction

**Figure 1:**
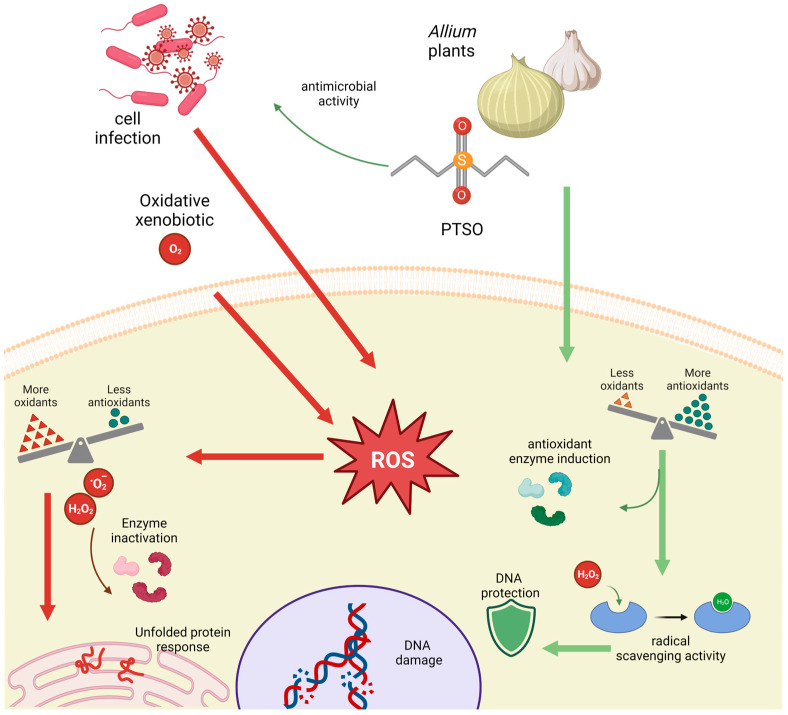
Scheme of PTSO activity in the cells (created with BioRender.com, accessed on 4 March 2024).

Organosulfur compounds (OSCs) are chemical molecules that contain sulfur atoms bound to carbon atoms [1]. Edible *Allium* plants, such as garlic (*Allium sativum*) or onions (*Allium cepa*), are rich sources of several OSCs [2,3]. These compounds are responsible for the characteristic pungent smell and taste of *Allium* vegetables [4]. *Allium* plants extracts are well known for their antioxidant properties [5,6,7], helping to neutralize toxic free radicals and reduce oxidative stress in the organism [8,9,10]. Among the OSCs of the *Allium* genus are the thiosulfonates, generated as secondary metabolites by alliinase [11]. Some of these thiosulfonates have high antioxidant and antibacterial properties demonstrated in a multitude of in vitro and in vivo studies [12,13,14,15,16]. Yin et al. [17] suggested that incorporating OSCs into food systems through exogenous addition could be a viable and advantageous approach for antioxidant protection. Several studies have provided evidence of the antioxidant potential of OSCs derived from *Allium* species, as they exhibit radical-scavenging activity and regulate the activity of cellular antioxidant enzymes [17,18,19,20]. Propyl-propane thiosulfonate (PTSO) is one of the secondary metabolites that arise mainly by decomposition of propiine in onion. PTSO has been considered for application in food packaging due to its potential to counteract the oxidative effects induced by H_2_O_2_, as observed in the reduction of reactive oxygen species (ROS) levels (Figure 1) [21].

Due to these characteristics, it has become a potential natural additive that can be used in nutrition, especially in animal nutrition, and as a biopesticide [22]. Over the past decade, there has been a notable increase in patents and research exploring the application of PTSO as a technological additive. As part of this trend, PTSO and another analogue, propyl-propane thiosulfinate (PTS), have been utilized to mitigate apicomplexa in animals [23]. Similarly, the effectiveness of PTSO and PTS has been demonstrated in preventing and reducing parasites in aquatic animals. These substances have also contributed to the mitigation of residues generated by antiparasitics and antibiotics, thereby assisting in environmental preservation [24].

In this sense, it is necessary to evaluate the toxicity of PTSO. The basic set of toxicological assessment includes studies on genotoxicity and subchronic oral toxicity. Both studies have been already conducted indicating safe potential use of PTSO at doses of 55 mg/kg [25,26]. PTSO is commonly utilized at a concentration of 0.3 mg/kg in feed, as indicated by Guillamón et al. [22]. Thus, in the case that a farm animal weighing 100 kg consumed 20 kg of feed per day, this would result in an exposure to PTSO of approximately 0.06 mg/kg body weight. It is important to note that this dose is significantly lower than those employed in toxicology tests. Recently, an in vivo study has been performed using adult mice exposed to the same doses of PTSO (in a range of 14–55 mg/kg b.w.) for 11 weeks [27], focusing on the estrous cycle, serum hormone levels, gene expression changes, and molecular docking in silico analysis. No major adverse effects were shown for all these endpoints. Despite all these promising results, further toxicological studies could be useful in the safety assessment, considering that other authors have indicated that *Allium* compounds may adversely affect testicular functions, leading to testosterone production inhibition, and exhibit spermicidal effects on spermatozoa [28]. 

In certain instances, elevated doses of antioxidant agents may manifest prooxidant effects [29]. Male infertility is influenced by various endogenous and exogenous factors, including oxidative stress, which is estimated to be present at elevated levels in up to 80% of infertile men, impacting several physiological pathways [30]. Since the late 20th century, the association between oxidative stress and male infertility has been extensively researched, leading to the introduction of new terms such as male oxidative stress infertility, proposed as a category for infertile men with high oxidative stress levels [31]. In this context, a comprehensive safety evaluation of antioxidant compounds at elevated doses, such as PTSO, is imperative to substantiate their safety concerning reproductive toxicology and their potential impacts across multiple generations.

Therefore, in order to complete the safety assessment, a two-generation reproductive toxicity study in mice fed with PTSO was performed for the first time. This study was conducted according to the OECD Guideline 416 [14]. Male and female CD1 mice were exposed to three dose levels of PTSO to investigate its potential toxics effects on two generations, focusing on F1 generation, and taking into account fertility and endocrine endpoints. To achieve this objective, body weight and food consumption were measured throughout the study for all generations (F0, F1, F2). Moreover, sexual hormone levels (progesterone -P-, testosterone -T-, estradiol -E2-, follicular stimulating hormone -FSH-, and luteinizing hormone -LH-) were measured in serum of the F1 generation. Likewise, an analysis of the sperm parameters and morphology were performed in the parental F0 and F1 generations. Finally, the organs were weighed and examined after necropsy for the histopathological study.

## 2. Materials and Methods

### 2.1. Animals, Housing, Feeding, and Environmental Conditions

A two-generation toxicity study was performed following the OECD Guideline 416 [32]. Eighty male and eighty female CD1 (Swiss) mice, both sexually mature and virgin, were ordered for this study (Charles River laboratories, Saint-Germain-Nuelles, France) to compose the F0 generation. The animals were 5 weeks old. Mice were chosen considering the advantages they represent versus the use of rats: the high number of animals needed in this experiment, their smaller size, reduced costs, and their higher fertility rates and shorter reproductive cycles [27,33,34]. All animals received human care in agreement with the Directive for the protection of animals used for scientific purposes (Directive, 2010/63/UE, Decision 2020/569/UE and Real Decreto, 2018), and all procedures were authorized by the Ethical Animal Experimentation Committee of the University of Córdoba and by the Junta de Andalucía (project no. 26–06-2018-104).

Although PTSO can be found naturally in species of the genus *Allium*, in this work it was synthesized chemically following the method described by Llana-Ruiz-Cabello et al. [21] and supplied by DMC Research Center. The PTSO was incorporated into estrogen-free feed, and pellets were created to compose the mice’s diet by a specialized company in supplemented feed (ROD14IRR, Altromin, Lage, Germany). Over the course of the study, and as the mice experienced weight gain, the concentration of PTSO in the feed was adjusted to guarantee the accurate intake of the selected trial doses. PTSO intake calculated for each dose in the F0 and F1 generations of male and female mice is presented in Figure S1. The environmental conditions of the animals were: temperature of 22 ± 3 °C, relative humidity of 50–60%, and a 12 h light/dark cycle.

### 2.2. Study Design

**Figure 2:**
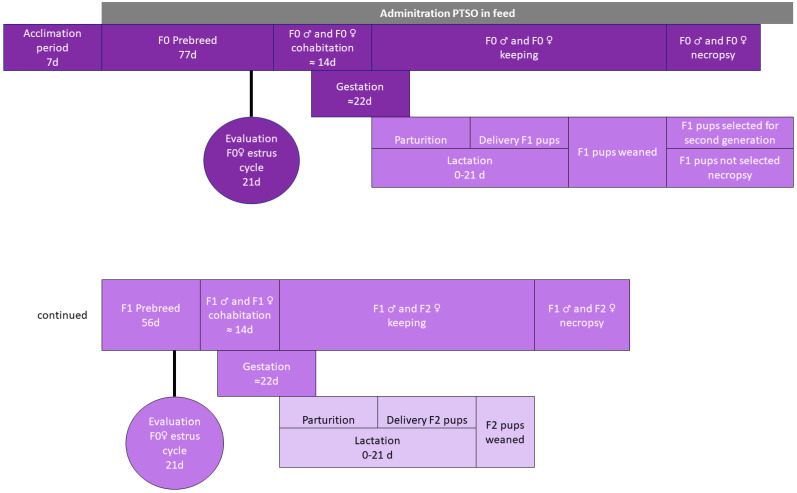
Design of the two-generation toxicity study.

The design of this experimental study is described in Figure 2. After 1 week for acclimation, with controlled conditions and periodical observations, mice were randomly divided into four groups: PTSO was administered through the diet in the 14, 28, and 55 mg PTSO/kg b.w./day dose groups, while the control group was exclusively fed an estrogen-free laboratory diet without PTSO. These selected doses were based on previous studies carried out in our laboratory in rats [21,26] and in mice [27]. PTSO was administered through the diet, while the control group was exclusively fed an estrogen-free laboratory diet. At the beginning of PTSO administration, mice were 6 weeks old. Following 77 days (11 weeks) of exposure to PTSO through the diet (PTSO was incorporated into the animal feed as explained above), the parental animals were paired for mating. Each mating pair underwent daily scrutiny for conclusive signs of mating, which were verified by the presence of a vaginal copulatory plug or the detection of sperm in a vaginal lavage. This was considered as gestation day 0. Once the F1 generation was born, they were kept exposed to PTSO or control feed until weaning. Moreover, 20 animals from the F1 generation by sex and group were selected to constitute the new parental generation. This new generation (parental F1) was subjected to the same procedures as the previous generation, except for being exposed for 56 days (8 weeks) to PTSO (premating period). F1 and F2 generation pups were exposed to PTSO during lactation and terminated at weaning. 

All animals successfully completed the entire treatment regimen. Subsequently, the mice underwent an overnight fast (18 h before euthanasia) and were deeply anesthetized with isoflurane. They were then humanely euthanized using CO_2_ and exsanguinated via intracardiac injection in accordance with Directive 2010/63/EU. 

### 2.3. In-Life Data Collection

For the parental generations F0 and F1 (*n* = 160/generation), body weight, body weights gain, and food consumption were measured weekly during the entire experiment (except during cohabitation). Individual clinical observations and signs of morbidity were recorded daily. Total food intake, feed conversion ratio, and feed efficiency were calculated for the entire exposure for parental F0 and F1, in premating, gestation, and lactation periods, when applicable.

$$Feed\;conversion\;ratio\;(FCR)=\frac{food\;intake\;(g)}{body\;weight\;gain\;(g)}$$

$$Feed\;efficiency\;(FE)=\frac{body\;weight\;gain\;(g)}{food\;intake\;(g)}\times 100$$

For the parental generations (F0 and F1) vaginal smears were obtained over a 5-day period in the week preceding the mating phase. In this procedure, a cotton-tipped swab was gently inserted into the vagina of the restrained mice and rotated to collect cells from the vaginal wall. The collected cells were transferred onto a dry glass slide by rolling the swab, after which they were air-dried. Subsequently, a Diff-Quik stain was applied. The prepared slides were then covered and immediately examined under bright-field illumination at 10× and 40× magnification. The determination of the estrous cycle phase was based by observation of leukocytes, cornified or nucleated epithelial cells, following the method described by Felicio et al. [33]. 

Reproductive performance in both males and females was assessed through the computation of the following metrics: % of non-gravid and gravid, mating, fertility, and conception indices (%), estrous cycle length, nº of implantation sites (all of them for the parental F0 and F1 generations), according to Tyl et al. [34].

$$Mating\;index\;(\%)=\frac{nº\;sperm\;or\;plug\;positive\;females}{nº\;females\;paired}\times 100$$

$$Fertility\;index\;(\%)=\frac{nº\;pregnant\;females}{nº\;sperm\;or\;plug\;positive\;females}\times 100$$

$$Conception\;index\;(\%)=\frac{nº\;females\;with\;live\;litters}{nº\;pregnant\;females}\times 100$$

For newborn pups (F1 and F2 generations), the following parameters were calculated [16]: % post-implantation loss/litter, live litter size, % still birth index/litter, % live birth index/litter.

$$\%\;Post-implantation\;loss\;per\;litter=\frac{nº\;uterine\;implantation\;sites-total\;nº\;pups\;on\;pnd\;0}{nº\;uterine\;implantation\;sites}\times 100$$

$$\%\;Still\;birth\;index\;per\;litter=\frac{nº\;pups\;dead\;on\;pnd\;0}{nº\;total\;pups\;on\;pnd\;0}\times 100$$

$$\%\;Live\;birth\;index\;per\;litter=\frac{nº\;live\;pups\;on\;pnd\;0}{nº\;total\;pups\;on\;pnd\;0}\times 100$$

At onset of parturition, the newborn pups (F1 and F2 litters) underwent an assessment for evident malformations, or alterations in behavior. On post-natal days (pnd) 0, 4, 7, 14, and 21, comprehensive physical examinations were conducted for every individual pup, and they were weighed. 

### 2.4. Serum Sex Hormone Levels

After sacrifice, collected blood samples were centrifuged to obtain the serum. The assessment encompassed the quantification of the subsequent serum hormones’ concentrations: progesterone -P-, testosterone -T-, estradiol -E2-, follicular stimulating hormone –FSH-, and luteinizing hormone -LH-. To accomplish this, the guidelines provided in the manufactured kits as detailed by Cascajosa-Lira et al. [27] and Casas-Rodriguez et al. [35] were followed.

### 2.5. Sperm Anlaysis

Following the OECD Guideline 416 [32], the control and high dose groups were analyzed first; if no treatment-related effects were seen, the other groups were not analyzed.

#### 2.5.1. Sperm Collection

Processing was performed with all media, dishes, slides, and microscopic plates at 37 °C. The epididymides were obtained immediately after mice from parental generations (F0, F1) were euthanized. Each epididymis was opened with scissors and the sperm mass was placed into a 100 µL droplet of Hank’s balanced salt solution (HBSS, Merck scientific, Rahway, NJ, USA), where it was cut with a blade and allowed to rest for 3 min, before 80 µL of the 100 µL droplet was aspirated and placed in a 1.5 mL Eppendorf tube. After homogenization, 10 µL of this suspension was diluted in 250 µL of HBSS supplemented with 4 mg/mL bovine serum albumin (BSA, Merck, Rahway, NJ, USA) and 0.33 mM sodium pyruvate.

#### 2.5.2. Computer-Assisted Sperm Motility Analysis (CASA)

For sperm concentration assessment and motility analysis, 2 µL of each diluted semen sample were placed in a Leja slide (Leja, 20 micron, Microptic, Barcelona, Spain). Ten random microscopic fields were analyzed in each replicate. The average concentration (×106 sperm /mL) was calculated by the SCA CASA software (Sperm class analyzer 6.5.0.91). To calculate the recovered sperm from the cauda epididymis, the concentration obtained in CASA was multiplied by the dilution factor.

The trajectory of each individual sperm was determined by the SCA CASA (Microptic, Barcelona, Spain) software (Sperm class analyzer 6.5.0.91) obtaining CASA sperm kinematic parameters: sperm curvilinear velocity (VCL, µm/s), straight-line velocity (VSL, µm/s), average path velocity (VAP, µm/s), linearity (LIN, VSL/VCL × 100), straightness (STR, VSL/VAP × 100), amplitude of lateral head displacement (ALH, µm) and beat-cross frequency (BCF, Hz) were assessed. Sperm morphology was calculated by visual observation of the CASA recordings. At least 200 sperm were counted. The percentage of morphologically normal sperm, abnormal heads, abnormal midpieces, and abnormal flagella were recorded in each sample.

### 2.6. Necropsy and Pathology

Following sacrifice, mice from the control and high dose groups (55 mg PTSO/kg b.w./day), underwent a comprehensive pathological examination during necropsy. For the histological evaluation of parental F0 and F1, the liver, kidney, spleen, brain, hypophysis, adrenal and thyroid glands, testicles, epididymis, seminal vesicles, prostate, uterus, and ovary were processed according to the protocol established in Cascajosa-Lira et al. [36]. For F1 and F2 offspring, the studied organs were spleen, brain, thymus, testicles, epididymis, uterus, and ovary. Testicles were preserved in Bouin’s fixative.

### 2.7. Statistical Analysis

The data are presented as mean values along with their corresponding standard deviations (SD) except for the sperm data, in which values are expressed as mean ± standard error of the mean (SEM). Statistical analyses were conducted using GraphPad Prism 9 software (GraphPad Software Inc., La Jolla, CA, USA) through one-way analysis of variance (ANOVA). The normality assumption was assessed using the Kolmogorov–Smirnov test. When statistically significant differences were observed, comparisons were performed using either the Tukey–Kramer multiple comparisons test or the Kruskal–Wallis test, followed by Dunn’s multiple comparison test in cases of non-normal data distribution. Statistical significance was considered when *p* < 0.05.

## 3. Results and Discussion

For the first time, a two-generation reproductive toxicity study in a mouse model has been carried out on a natural organosulfur compound from the genus *Allium*, PTSO. During the study, only two female animals died: specifically, animal number 237 from the control group (0 mg PTSO/kg b.w./day) and animal number 272 from the low dose level (14 mg PTSO/kg b.w./day). These fatalities occurred prior to giving birth and were related to birth-related causes. No relationship between these deaths and the administration of PTSO could be stablished. Furthermore, some offspring died as a result of cannibalism by the mothers. No significant clinical observations were noted throughout the study.

### 3.1. In-Life Data Report

**Figure 3:**
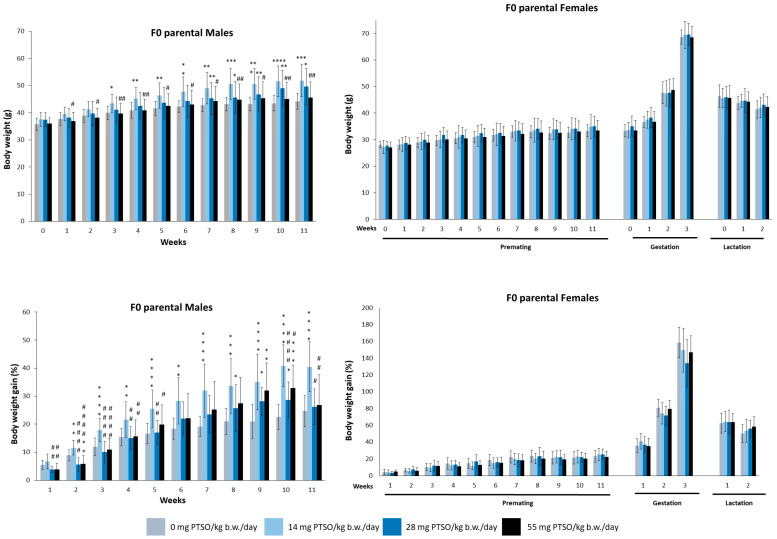
Mean body weight (g) and body weight gain (%) of male and female parental F0 mice orally exposed to 0, 14, 28, and 55 mg PTSO/kg b.w./day for the whole experiment. Values are mean ± standard deviation (SD) for 20 mice/sex/group. (* *p* < 0.05, ** *p* < 0.01, *** *p* < 0.001, **** *p* < 0.0001). *—Statistical difference compared with the respective control group. (# *p* < 0.05, ## *p* < 0.01, #### *p* < 0.0001). #—Statistical difference compared with 14 mg PTSO/kg b.w./day.

Body weight and body weight gain increased during the study period following a usual pattern in mice of parental F0 (Figure 3). Males fed 14 mg PTSO/kg b.w./day experienced statistical increases in their body weight mean from the third week. Moreover, between weeks 1 and 11, male animals in the 55 mg dose group were significantly lighter than those in the 14 mg PTSO/kg b.w./day group. In the case of body weight gain, the male animals also showed statistically significant differences. The 28 and 55 mg PTSO/kg b.w./day group showed significant differences (*p* < 0.01) from the first week compared to the 14 mg PTSO/kg b.w./day group. In addition, the 14 mg PTSO/kg b.w./day group started to show differences from the second week, growing significantly more than the control group. These results are in accordance with those obtained in other species. Other authors have observed an increased average daily weight gain (ADG) compared to the control group in piglets fed with 15 mg/kg of feed of PTSO [37]. Similarly, other authors have demonstrated a higher ADG in growing-finishing pigs fed with 30 mg/kg of feed of PTSO compared to those animals that received a control diet [37]. In contrast, females of this generation showed no significant differences in relation to body weight or body weight gain during all stages of the study (premating, gestation, and lactation). The metabolic demands of pregnancy and lactation in female rodents could also alter their energy balance and nutrient utilization, as suggested by Rivera et al. [38]. This factor might explain why females in the study did not exhibit the same weight gain patterns as their male counterparts.

**Figure 4:**
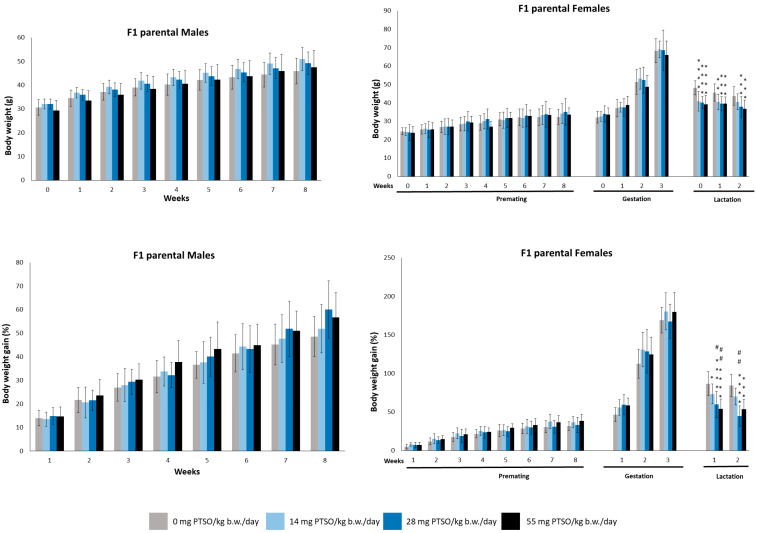
Mean body weight (g) and body weight gain (%) of male and female parental F1 mice orally exposed to 0, 14, 28, and 55 mg PTSO/kg b.w./day for the whole experiment. Values are mean ± standard deviation (SD) for 20 mice/sex/group. (* *p* < 0.05, ** *p* < 0.01, *** *p* < 0.001, **** *p* < 0.0001). *—Statistical difference compared with the respective control group. (# *p* < 0.05, ## *p* < 0.01). #—Statistical difference compared with 14 mg PTSO/kg b.w./day.

Similarly, the parental F1 generation grew at a normal rate for this species, as shown in Figure 4. Significant differences were found in female animals in this generation, but only during the lactation period. Regarding body weight, all dose groups showed significant decreases with respect to the control group. These significant differences remained from the first to the third week of lactation. The body weight gain of parental F1 females also showed significant differences during lactation: the first week, the animals of all dose groups showed significative differences compared to the control group, and the 28 and 55 mg PTSO/kg b.w./day dose groups showed significant differences compared to the 14 mg PTSO/kg b.w./day dose group. During the second week of lactation, significant reductions in body weight gain were observed, evident exclusively in the groups administered with 28 mg PTSO/kg b.w./day and 55 mg PTSO/kg b.w./day in comparison to the control group. Additionally, the 28 mg PTSO/kg b.w./day group exhibited statistically significant differences when compared with the 14 mg PTSO/kg b.w./day group. In our research, we observed a slight weight loss during lactation in F1 female mice compared to F0 generation. This fact could be due to higher energy-demanding processes during lactation [39]. Furthermore, alliaceous compounds such as PTSO and its precursor PTS are known for their anti-obesity effects in obese mice, as reported by other authors [22,37], which could explain this weight reduction observed in certain murine models.

**Figure 5:**
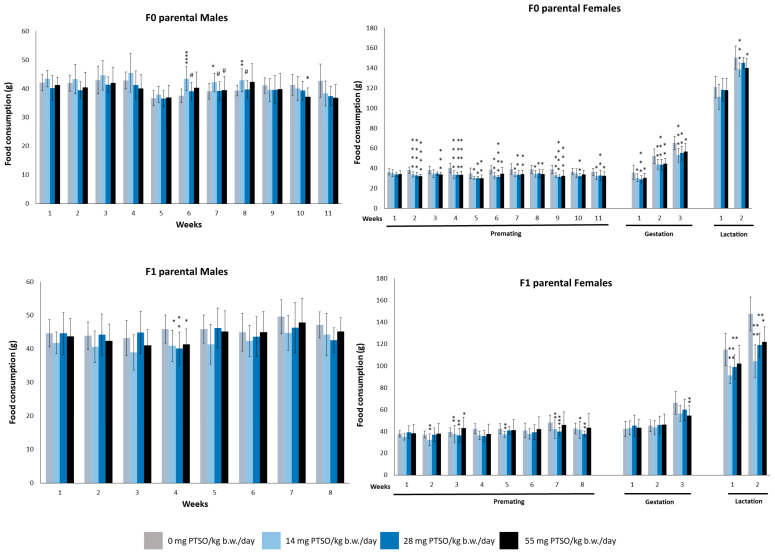
Food consumption (g) of male and female parental F0 and F1 mice orally exposed to 0, 14, 28, and 55 mg PTSO/kg b.w./day for the whole experiment. Values are mean ± standard deviation (SD) for 20 mice/sex/group. (* *p* < 0.05, ** *p* < 0.01, *** *p* < 0.001, **** *p* < 0.0001). *—Statistical difference compared with the respective control group. (# *p* < 0.05). #—Statistical difference compared with 14 mg PTSO/kg b.w./day.

Feed consumption for both parental F0 and F1 generations is represented in Figure 5. The feed consumption showed certain significant differences, especially in female animals. For male mice from the F0 generation, these differences became apparent starting at week 6 but gradually disappearing until week 10. F1 parental male animals showed significant differences only in the fourth week for all dose groups. Females of the F0 generation showed variations in feed consumption in all dose groups during premating, gestation, and lactation, except for the first week of lactation. Similarly, F1 generation female animals displayed variations compared to control groups in several weeks across all dose groups, although these were not consistent and appeared sporadically throughout the premating, gestation, and lactation periods. After the end of each study period (premating, management, and lactation) the total feed intake, feed conversion ratio (FCR), and feed efficiency (FE) were calculated for parental F0 and F1 generations, and are represented in Table 1. FE shows some statistical differences in males from the F0 generation: the results of the dose groups of 14 and 28 mg PTSO/kg b.w./day were significantly higher when they were compared with those of the control groups. Regarding the total feed intake, the female animals of this generation showed significant lower values in all dose groups when compared to the control group during the premating and gestation periods but not during lactation. In contrast, both sexes of F1 generation showed no significant differences at any period. These significant differences in weight, intake and efficiency seem to have no clear relationship to the administration of PTSO in the feed. These differences appear exclusively in one of the two sexes and not in a dose-dependent manner. In previous studies in rats, PTSO did not produce alterations in animal weight or food or water consumption in a subchronic assay [26,40] during the 13 weeks of exposure. Consequently, the responses of mouse and rat model to dietary subchronic administration of PTSO have been similar. Studies have also been carried out in other species such as fish (*Sparus aurata*), where no alterations in the weight of the animals have occurred during the 12 weeks of the trial, even when using doses approximately three times higher (150 mg PTSO/kg b.w.) [26]. However, in an attempt to explain these findings, it might be hypothesized that the minor differences in consumption observed in female mice could be linked to their heightened sensitivity to palatability, especially since PTSO is a flavor compound known for its significant sensory impact. The study by Dahir et al. [41] provides substantial insights indicating that estradiol influences the observed sex differences in fat taste responsiveness, supporting the concept of heightened taste sensitivity in female mice. Aligning with this hypothesis, these findings imply that minor differences in consumption among female mice could stem from their increased sensitivity to palatable substances such as organosulfur *Allium* compounds. 

The reproductive parameters for all generations (F0, F1, and F2) are shown in Table 2. There were no significant differences in any of the parameters calculated for any generation. The mating, fertility, and conception indices show normal reproductive activity for this species. In the present work, the analysis of the length of the estrous cycle was normal and is in agreement with previous results obtained in other two-generation studies using CD1 mice [34]. Moreover, a cytological study on epithelial cells and leukocytes in mice exposed subchronically to PTSO had been previously carried out and no differences were evident [27] in accordance with the present work. The number of offspring per litter was normal for each of the dose groups and the vast majority was born alive. Therefore, taking into account all these results, PTSO showed no effect on reproduction and development of mice.

**Figure 6:**
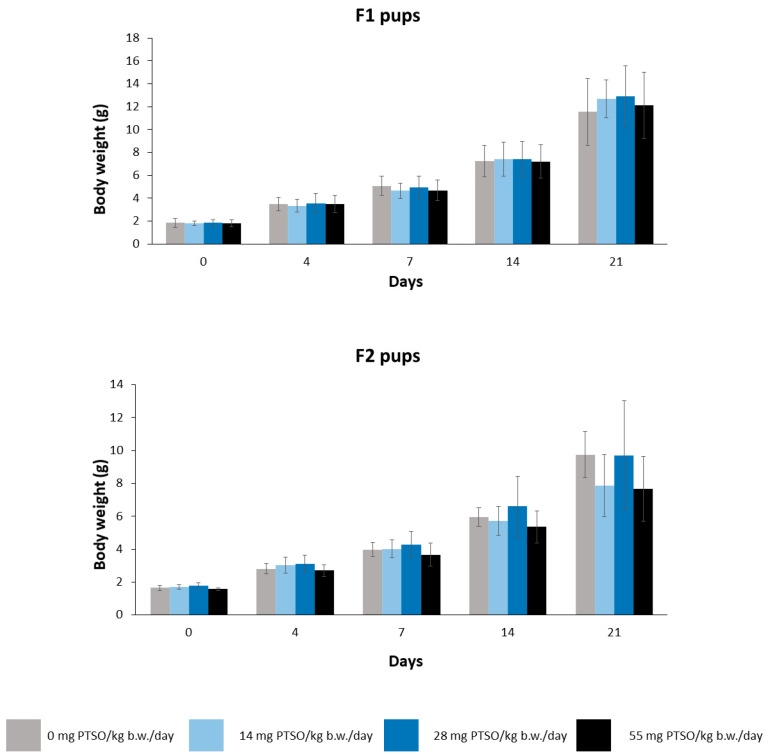
Mean body weight (g) of F1 and F2 pups orally exposed to 0, 14, 28, and 55 mg PTSO/kg b.w./day for 21 days. Values are mean ± standard deviation (SD) for 20 mice/sex/group.

Figure 6 shows the body weight of the F1 and F2 pups. There were no statistically differences at any of the doses tested for both generations. Consequently, pregnant mice exposed to PTSO are not likely to suffer damage that could affect the development of the offspring. Although there are limited studies delving into the influence of PTSO on fertility, several studies in other species have demonstrated a positive impact of PTSO intake on reproductive efficiency. For instance, in laying hens, an increase in both egg size and quantity was observed in animals fed with this compound [22].

### 3.2. Hormone Serum Levels

**Figure 7:**
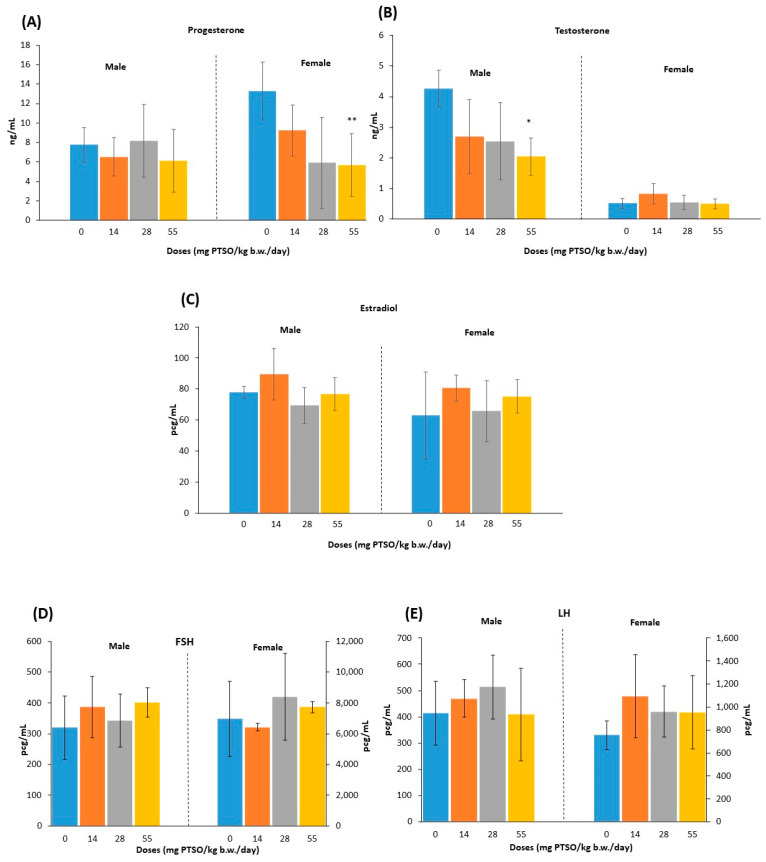
Serum concentrations (ng/mL or pcg/mL) of sexual hormones: (**A**) progesterone, (**B**) testosterone, (**C**) estradiol, (**D**) follicle stimulating hormone, and (**E**) luteinizing hormone. For (**D**) and (**E**), the left axis represents males, and the right axis represents females. (* *p* < 0.05, ** *p* < 0.01). *—Statistical difference compared with the respective control group.

Once the F1 generation was sacrificed, sex hormone levels were measured in serum. The serum concentrations of P, T, E2, FSH, and LH for male and female mice of F1 generation are represented in Figure 7. While there was no significant change in P levels in male mice, a decrease was observed in female mice at 55 mg PTSO/kg b.w./day when compared to the control group (*p* < 0.01). Importantly, this alteration is not of toxicological concern, as the observed P levels remained within the normal range as defined by Frye et al. [42]. Similarly, T levels showed a significant reduction in male mice at 55 mg PTSO/kg b.w./day when compared with the control groups (*p* < 0.05), although these levels were within a normal range [43]. In contrast, female mice showed no significant alteration in T levels. Certain studies have indicated that endocrine disruptors, which impact redox balance, can lead to a decrease in testosterone levels and influence sperm motility and morphology [44]. Nevertheless, it seems that PTSO does not exert any adverse effects on testosterone levels. No statistically significant differences were observed for E2 levels at any dose assayed for both male and female mice, and the concentrations detected in serum for both sexes are in the range of other studies [45,46]. In the same way, FSH and LH showed no significant differences at any dose tested in either sex. Consequently, PTSO did not produce alterations in sex hormone levels even when animals were exposed during their development in gestation, and subsequently for 13 weeks. These findings are in accordance with previous studies in which PTSO did not produce any alteration under similar exposure conditions [27].

### 3.3. Sperm Motility and Morphology

As shown in Table 3, Table 4 and Table 5, no significant differences (*p* > 0.05) were found between F1 control and high dose groups in any of the sperm parameters analyzed. Only some significant differences were found in the F0 group. 

While there were no significant differences in the number of recovered sperm among treatments (*p* > 0.05) in the F0 group, a decrease in total and progressive sperm motility was noted in mice exposed to medium and high doses of 28 and 55 mg/Kg b.w. (Table 3). It is important to note that, unlike the F0 group, the F1 mice were exposed to PTSO not only directly during their adult age but also indirectly during gestation and lactation. Therefore, it is not possible to establish a correlation between the differences found in the F0 group and exposure to the tested compound, PTSO, given that the F1 group was exposed for a longer duration and did not showed any alterations.

In fact, recent studies have highlighted the protective effects of garlic extract on sperm quality. Soleimanzadeh et al. [47] demonstrated that garlic extract significantly improved sperm quality in mice exposed to busulfan-induced testicular toxicity, including increased antioxidant activity and hormone levels. Similarly, El-Ratel et al. [48] found that dietary supplementation of garlic extracts in rabbit bucks led to improved semen quality, enhanced hepatic antioxidant activity, and increased reproductive hormone levels. In addition, onion crude extract has been reported to improve sperm quality, including motility in cadmium testicular toxicity-induced rats [49].

Similar results were obtained when analyzing the kinematic parameters (Table 4). Total and progressive sperm motility yielded significantly lower values on the sperm samples from F0 mice submitted to medium and high doses. Importantly, this decrease did not impact the fertility parameters evaluated in F0 mice as reflected in Table 2. Moreover, F1 mice exposed to the highest dose exhibited no significant changes in sperm motility compared to the control group. Sperm motility analysis is a valuable indicator when assessing the impact on sperm quality and motion, which is a requirement for fertility. According to Perreault and Cancel [50], prolonged exposure to a chemical agent or high concentrations can potentially lead to testicular atrophy, reducing sperm production and affecting fertility. In our study, the mild detrimental effect of medium and high dosages of the agent showed up as a decrease in the percentage of motile spermatozoa and the kinematic features only in F0 mice, with no significant impact on fertility parameters. It has been reported before that a particular effect may be observed only in the parental generation, particularly when the effect is on the border of statistical significance. These results could be interpreted as chance findings, not related to the test compound, despite the identical underlying biological effect [51]. In our study, this discrepancy may be attributed to the age and exposure time to the compound in both generations. Several studies have found that sperm quality becomes more susceptible to stressors as individuals age [52,53,54]. This occurred with no effect on any fertility parameters in any of the generation studied, possibly due to the small reduction on sperm motility parameters, even after exposure to high doses.

The lower sperm motility observed after the exposure to medium and high dosages in F0-mice could be explained by the analysis of sperm morphology. There was a significantly lower percentage of normal forms in the sperm from F0 mice exposed to medium and high dosages of 28 and 55 mg/Kg b.w. These changes were not observed in F1 mice (Table 5). Additionally, this decrease was not related to detrimental effects on the fertility parameters studied found in Table 2. Many authors have studied factors affecting sperm morphology [55,56]. However, most of these studies were focused on the sperm morphology in infertile or subfertile mice, so the relationship between changes in sperm morphology and normal fertility in males remains uncertain [57]. In our study, the increase in sperm tail abnormalities observed in F0 did not affect any of the fertility parameters evaluated, and no effect on sperm morphology or male fertility was found in F1 mice exposed to high doses of the compound. The greater effect in F0 mice could be due to a higher sensitivity in older mice [53] or a random finding [51]. 

Finally, genetic variability in the mice should also be considered, as genetic factors can significantly impact sperm motility [58]. 

In recent years, a significant association has been identified between the involvement of natural antioxidants, such as resveratrol, and enhanced gamete formation [59]. However, there is a lack of evidence-based antioxidant treatments directly improving seminal parameters or birth ratios, contributing to ongoing controversy over their utilization [31].

### 3.4. Necropsy, Organ Weights, and Histopathology

The absolute organ weights of parental F0, parental F1, and F1 and F2 offspring are shown in Table 6, Table 7 and Table 8, respectively. The organ weight/b.w. ratio and brain ratio of parental F0, parental F1, and F1 and F2 offspring are shown in Supplementary Materials (Tables S1, S2, and S3, respectively).

For the parental and offspring generations, no alterations in the value of absolute weights or in the value of ratios to body weight or brain weight were detected when compared with the control group. In agreement with this work, Cascajosa-lira et al. [11] found similar results in a subchronic 90-day assay with PTSO, where the rats did not show any alteration in the absolute organ weight or ratios.

**Figure 8:**
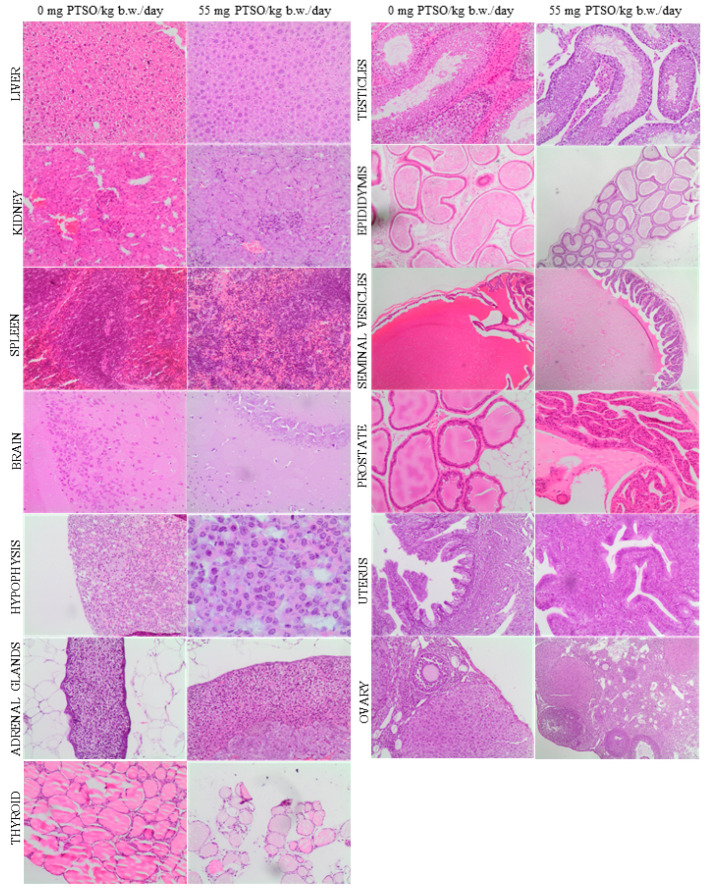
Representative histological images of the organs comparing both 0 and 55 mg PTSO/kg b.w./day in parental F0 mice. Liver, kidney, spleen, brain, hypophysis, adrenal and thyroid glands, testicles, epididymis, seminal vesicles, prostate, uterus, and ovary H&E stains (objective magnification = 100×).

**Figure 9:**
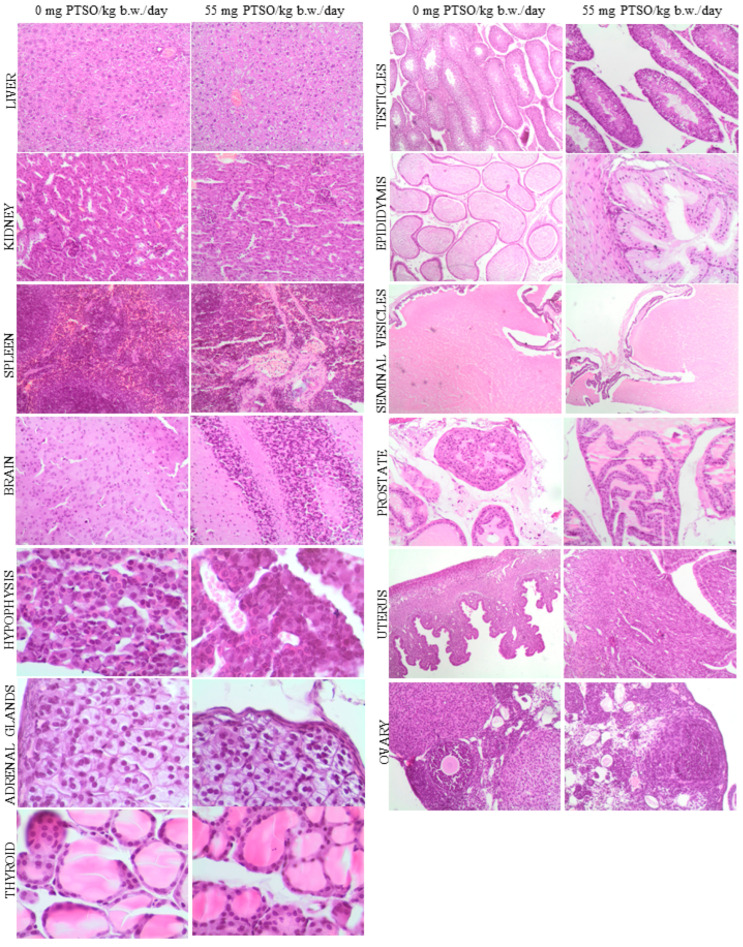
Representative histological images of the organs comparing both 0 and 55 mg PTSO/kg b.w./day in parental F1 mice. Liver, kidney, spleen, brain, hypophysis, adrenal and thyroid glands, testicles, epididymis, seminal vesicles, prostate, uterus, and ovary H&E stains (objective magnification = 100×).

**Figure 10:**
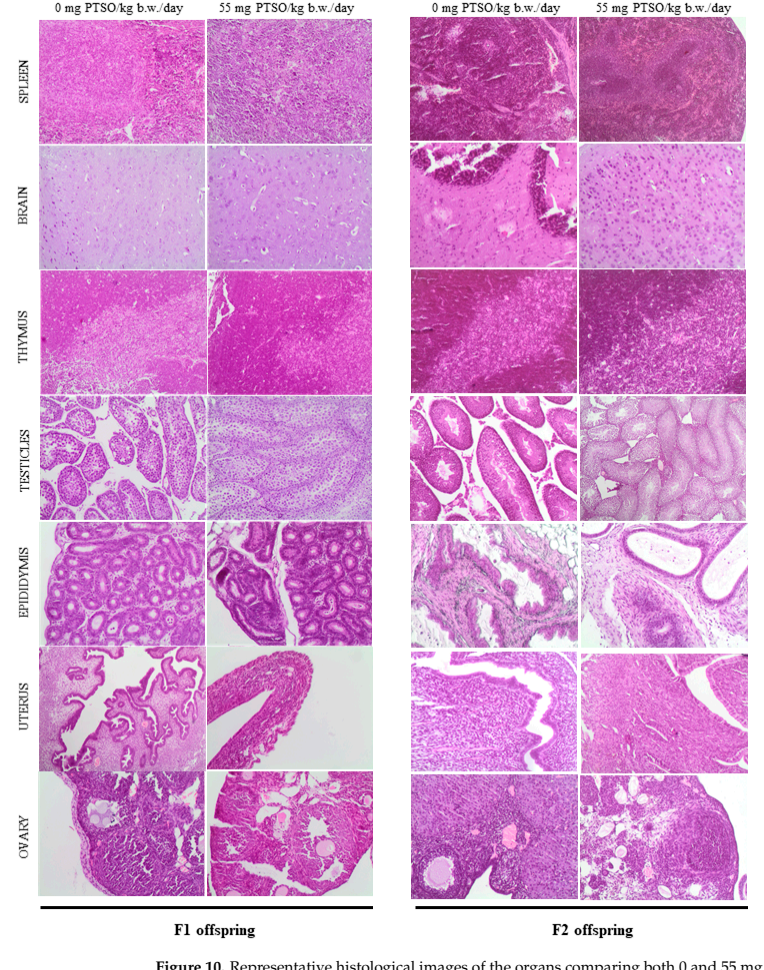
Representative histological images of the organs comparing both 0 and 55 mg PTSO/kg b.w./day in pups of F1 and F2 mice. Spleen, brain, thymus, testicles, epididymis, uterus, and ovary H&E stains (objective magnification = 100×).

The results obtained from the histopathological study of the analyzed samples from different organs did not show signs of pathology in the high dose groups (55 mg PTSO/kg b.w./day) in any generations studied, when compared to the control groups. The absence of tissue alterations was observed in parental F0 and F1 generations (Figure 8 and Figure 9, respectively), both in males and females, as well as in the offspring (F1 and F2) (Figure 10). In this sense, the liver exhibited a normal morphology, with hepatocytes showing a normal structure. The kidney of the parents (F0 and F1) did not show relevant alterations either at the glomerular and tubular level or in the renal interstitium (Figure 8 and Figure 9). No significant pathological lesions were observed in the spleen of the parents and offspring generations, including the red or white splenic pulp, in any of the studied groups. There were no relevant brain changes or pathological lesions observed in the brains of any treated groups of parents (F0 and F1) or offspring (F1 and F2). No relevant alterations were observed in the pituitary gland (adenohypophysis or neurohypophysis) in any treated parental groups compared to the control, nor were significant changes evident in these groups in the adrenal and thyroid glands. The thymus of both generations of offspring showed no relevant pathological lesions in the cortex or medulla (Figure 10). Regarding male reproductive organs, they exhibited normal histology in both parents and offspring. Specifically, no significant pathological lesions were observed in the testicles, including seminiferous tubules and interstitium. The epididymal ducts appeared to be normal, moderately dilated, and filled with spermatozoa. Similarly, the seminal vesicles and prostate showed normal structure in all treated groups from different generations. In the female reproductive organs of parents and offspring, no relevant pathological signs were observed. The uterus showed no relevant lesions at the endometrial level, and the ovaries exhibited an apparently normal structure of the ovarian epithelium, tunica albuginea, cortical and medullary regions, with no histopathological differences in mothers and offspring of all treated groups and generations compared to the control groups.

In a study conducted by Mellado-García et al. [25], it was observed that rats treated with 55 mg PTSO/kg b.w./day (administered three doses at 0, 24, and 45 h, with euthanasia at 48 h) exhibited minor damage in liver and stomach. In contrast, in the present study in mice administered the same dose level for a longer time, no histopathological lesions were detected. These differences can be attributed to the method of exposure employed in the present study, which involved the feed as a vehicle for PTSO, instead of oral gavage in the previous trials, which used a stomach tube after a fasting period. In the present work, the incorporation of the test item in the feed may have had a less detrimental impact on the gastrointestinal system due to reduced direct contact. In addition, the exposure of the additive through the feed better resembles a real scenario of consumer exposure. The results are similar to those obtained in previous subchronic studies where no histological damage was found [26,40].

### 3.5. Final Remarks

The safety of an additive intended to be used use in animal nutrition should be assessed on the basis of the toxicological studies performed in vitro and in vivo on laboratory animals [60]. In order to complete the safety assessment of PTSO, a two-generation reproductive toxicity study in mice fed with this antioxidant compound was performed for the first time, providing further toxicological information. 

When comparing the findings achieved in the present two-generation study in CD-1 mice exposed to PTSO for 20 weeks (F0 generation) with those previously obtained in Sprague-Dawley rats exposed to 13 weeks in the subchronic research, both experimental models have shown similar sensitivity to PTSO, without producing toxic effects in both species. Therefore, a NOAEL of ≥ 55 mg PTSO/kg b.w./day can be established for both parental and offspring animals. These studies conducted in mice and rats, following regulatory testing guidelines, play a crucial role in risk assessment. They are robust, examining a wide array of relevant and sensitive parameters to identify any potential adverse outcomes. These studies cover a broad spectrum of doses, administering substances via a relevant route of exposure during sensitive life stages, spanning multiple generations in appropriate animal models, and incorporating validated endpoints. Moreover, they involve large numbers of animals per group, ensuring comprehensive and reliable data for risk evaluation [34].

## 4. Conclusions

In conclusion, the safety assessment of the antioxidant compound PTSO for its use in animal nutrition has been enriched by the performance, for the first time, of a two-generation reproductive toxicity study in CD-1 mice. This study has revealed no toxic effects, regarding body and organ weights, reproductive, teratogenic, or hereditary aspects, serum hormone levels, and histopathological observations. Consequently, the NOAEL of ≥ 55 mg PTSO/kg b.w./day can be established for both parental and offspring animals. The incorporation of a substantial number of animals used and parameters calculated in the present work enhances the reliability and robustness of the data, providing a solid foundation for the comprehensive evaluation of PTSO’s safety profile in the context of animal nutrition.

## Figures and Tables

**Table 1:** Total food intake, feed conversion ratio, and feed efficiency in CD1 mice fed with different doses of PTSO (0, 14, 28, and 55 mg/kg b.w./day). Values are mean and SD for 20 mice/sex/group. (* *p* < 0.05, ** *p* < 0.01, *** *p* < 0.001). The coded symbols represent the following: *—Statistical differences compared with the respective control group.

		Doses (mg PTSO/kg b.w./day)			
		0	14	28	55
**F0 male**					
**Premating period**	Total food intake (g)	448.93 ± 28.31	459.37 ± 41.45	433.40 ± 32.05	429.52 ± 40.07
	Feed conversion ratio (FCR)	53.03 ± 9.56	32.95 ± 11.78	39.91 ± 13.43	48.29 ± 19.62
	Feed efficiency (FE)	1.95 ± 0.41	3.38 ± 1.11 ***	2.80 ± 0.99 *	2.43 ± 1.01
**F0 female**					
**Premating period**	Total food intake (g)	407.16 ± 37.66	367.22 ± 23.58 *	367.49 ± 21.69 *	365.11 ± 11 *
	Feed conversion ratio	58.87 ± 13.99	56.00 ±18.78	52.62 ± 16.40	66.02 ± 21.03
	Feed efficiency (FE)	1.79 ± 0.45	1.90 ± 0.60	2.08 ± 0.71	1.70 ± 0.53
**Gestation period**	Total food intake (g)	152.61 ± 14.17	128.42 ± 5.18 *	125.71 ± 20.26 **	126.50 ± 9.97 **
	Feed conversion ratio (FCR)	4.45 ± 0.60	3.73 ± 0.67	3.64 ± 0.95	3.62 ± 0.39
	Feed efficiency (FE)	22.83 ± 3.04	27.48 ± 4.53	28.82 ± 6.24	27.94 ± 3.28
**Lactation period**	Total food intake (g)	267.33 ± 17.53	251.40 ± 13.77	263.90 ± 9.39	263.64 ± 12.54
	Feed conversion ratio (FCR)	−49.44 ± 19.34	−45.18 ± 24.34	−51.11 ± 20.55	−40.91 ± 15.88
	Feed efficiency (FE)	−2.39 ± 1.25	−2.73 ± 1.20	−2.25 ± 0.87	−2.78 ± 1.11
**F1 male**					
**Premating period**	Total food intake (g)	366.68 ± 32.19	335.57 ± 82.30	395.77 ± 68.38	345.37 ± 56.39
	Feed conversion ratio (FCR)	25.24 ± 6.28	20.08 ± 7.05	22.09 ± 6.89	20.11 ± 5.80
	Feed efficiency (FE)	4.24 ± 1.21	5.55 ± 1.87	4.92 ± 1.37	5.38 ± 1.53
**F1 female**					
**Premating period**	Total food intake (g)	329.19 ± 31.38	303.66 ± 34.86	307.60 ± 26.92	367.13 ± 107.49
	Feed conversion ratio (FCR)	44.42 ± 10.78	39.29 ± 12.94	36.76 ± 12.93	40.70 ± 10.97
	Feed efficiency (FE)	2.35 ± 0.45	2.76 ± 0.73	3.02 ± 0.95	2.64 ± 0.74
**Gestation period**	Total food intake (g)	149.95 ± 20.42	147.38 ± 19.02	153.15 ± 19.67	149.80 ± 42.42
	Feed conversion ratio (FCR)	4.14 ± 0.83	4.17 ± 0.85	4.34 ± 0.87	4.12 ± 1.06
	Feed efficiency (FE)	25.42 ± 7.08	24.77 ± 4.19	24.44 ± 7.79	25.49 ± 5.34
**Lactation period**	Total food intake (g)	232.00 ± 51.67	198.25 ± 21.01	199.25 ± 45.99	214.71 ± 29.16
	Feed conversion ratio (FCR)	−43.15 ± 18.67	−44.35 ± 35.10	−48.31 ± 15.74	−63.54 ± 30.25
	Feed efficiency (FE)	−2.78 ± 1.29	−3.61 ± 2.25	−2.29 ± 0.77	−1.98 ± 1.04

**Table 2:** Reproductive performance parameters in F0, F1, and F2 generation of CD1 mice fed with different doses of PTSO (0, 14, 28, and 55 mg/kg b.w./day). N = 20 mice/sex/group.

	Dose (mg PTSO/kg b.w./day)											
	F0				F1				F2			
	0	14	28	55	0	14	28	55	0	14	28	55
**Non-gravid (%)**	0.00	0.00	5.00	10.00	5.00	5.00	5.00	0.00				
**Gravid (%)**	100.00	100.00	95.00	90.00	95.00	95.00	95.00	100.00				
**Mating index (%)**	100.00	100.00	100.00	90.00	100.00	95.00	95.00	100.00				
**Fertility index (%)**	100.00	100.00	95.00	100.00	95.00	100.00	100.00	100.00				
**Conception index (%)**	100.00	100.00	100.00	100.00	100.00	100.00	100.00	100.00				
**Estrous cycle length** **(mean days ± SD)**	4.69 ± 0.60	5.00 ± 00	4.90 ± 0.32	4.82 ± 0.40	4.79 ± 0.558	4.94 ± 0.24	4.53 ± 0.62	4.61 ± 0.78				
**Nº of implantation sites** **(mean ± SD)**	14.33 ± 1.85	14.56 ± 3.33	15.28 ± 1.96	15.11 ± 2.72	15.00 ± 1.97	14.47 ± 2.61	13.80 ± 4.61	14.55 ± 5.43				
**% PIL (mean ± SD)**					12.45 ± 9.80	11.08 ± 5.90	11.24 ± 8.05	12.69 ± 9.46	6.45 ± 5.92	8.37 ± 7.72	8.94 ± 6.85	7.61 ± 5.22
**LLS (mean ± SD)**					12.00 ± 3.35	13.16 ± 2.81	12.63 ± 3.17	13.80 ± 3.81	14.16 ± 1.92	13.58 ± 2.50	12.84 ± 4.09	14.53 ± 2.58
**% LBI (mean ± SD)**					0.00 ± 0.00	0.00 ± 0.00	0.00 ± 0.00	0.00 ± 0.00	0.00 ± 0.00	0.00 ± 0.00	0.00 ± 0.00	1.56 ± 6.99
**% SBI (mean ± SD)**					100.00 ± 0.00	100.00 ± 0.00	100.00 ± 0.00	100.00 ± 0.00	100.00 ± 0.00	100.00 ± 0.00	100.00 ± 0.00	98.44 ± 6.99

LBI: live birth index per litter; LLS: live litter size; PIL: post-implantation loss per litter; SBI: still birth index per litter.

**Table 3:** Sperm parameters assessed by computer-assisted sperm analysis obtained from the cauda epididymis of parental F0 and F1 fed with different doses of PTSO (0, 14, 28, and 55 mg/kg b.w./day).

	Recovered Sperm (10^6^ Sperm/mL)	Total Motility (%)	Progressive Motility (%)
**F0–Control**	13.61 ± 0.96	74.82 ± 3.27	43.34 ± 4.14
**F0—14 mg/kg b.w./day**	14.02 ± 1.36	61.36 ± 3.36	32.51 ± 3.41
**F0—28 mg/kg b.w./day**	13.84 ± 1.08	59.24 ± 3.24 *	27.05 ± 1.28 *
**F0—55 mg/kg b.w./day**	13.41 ± 0.85	66.88 ± 3.10 *	30.07 ± 1.80 *
***p* value**	*p* = 0.980	*p* = 0.008	*p* = 0.002
**F1–Control**	9.81 ± 0.66	58.55 ± 4.38	33.39 ± 3.87
**F1–55 mg/kg b.w./day**	10.46 ± 0.84	55.70 ± 2.89	32.40 ± 3.20
***p*-value**	*p* = 0.552	*p* = 0.593	*p* = 0.846

Values are mean ± standard error of the mean (SEM). * Significant differences in comparison with the respective control group.

**Table 4:** Sperm kinematics obtained by computer-assisted sperm analysis (CASA) in parental F0 and F1 fed with different doses of PTSO (0, 14, 28, and 55 mg/kg b.w./day).

	VCL (µm/s)	VAP (µm/s)	VSL (µm/s)	STR (%)	LIN (%)	ALH (µm)	BCF (Hz)
**F0–Control**	150.83 ± 12.57	67.45 ± 6.01	50.11 ± 4.90	60.94 ± 1.32	27.10 ± 0.90	4.96 ± 0.35	15.98 ± 0.33
**F0—14 mg/kg b.w./day**	137.09 ± 9.44	61.28 ± 4.53	43.66 ± 3.83	58.56 ± 1.50	26.00 ± 0.93	4.65 ± 0.26	14.92 ± 0.43
**F0—28 mg/kg b.w./day**	111.62 ± 6.24 *	48.93 ± 3.37 *	35.21 ± 3.17 *	57.28 ± 2.22	25.30 ± 1.72	3.93 ± 0.19 *	15.38 ± 0.23
**F0—55 mg/kg b.w./day**	118.43 ± 6.05	50.67 ±2.75 *	35.53 ± 2.17 *	56.15 ± 1.06	23.35 ± 0.64	4.12 ± 0.18	15.00 ± 0.28
***p*** **–value**	*p* = 0.016	*p* = 0.013	*p* = 0.018	*p* = 0.186	*p* = 0.135	*p* = 0.023	*p* = 0.109
**F1–Control**	143.83 ± 8.46	67.11 ± 4.02	46.74 ± 3.74	58.83 ± 1.53	26.17 ± 0.91	4.76 ± 0.22	15.74 ± 0.42
**F1–55 mg/kg b.w./day**	151.96 ± 12.51	70.03 ± 5.62	50.93 ± 4.41	60.34 ± 1.87	28.38 ± 1.13	5.08 ± 0.36	15.88 ± 0.40
***p*** **-value**	*p* = 0.597	*p* = 0.402	*p* = 0.477	*p* = 0.526	*p* = 0.144	*p* = 0.457	*p* = 0.811

VCL = curvilinear velocity; VAP = velocity of the average path; VSL = straight-line velocity; STR = straightness (VSL/VAP × 100); LIN = linearity (VSL/VCL × 100); ALH = amplitude of the lateral head displacement; BCF = beat-cross frequency. * Significant differences in comparison with the respective control group.

**Table 5:** Effect of PTSO on the sperm morphology of parental F0 and F1 fed with different doses of PTSO (0, 14, 28, and 55 mg/kg b.w./day).

	Normal Forms	Ab. Head	Ab. Midpiece	Ab. Tail
**F0—Control**	66.99 ± 1.49	1.75 ± 0.36	16.04 ± 1.93	15.22 ± 1.75
**F0—14 mg/kg b.w./day**	66.08 ± 0.89	1.51 ± 0.21	16.44 ± 1.14	15.97 ± 1.02
**F0—28 mg/kg b.w./day**	60.19 ± 1.39 *^#^	2.15 ± 0.50	19.03 ± 1.38	19.64 ± 1.57
**F0—55 mg/kg b.w./day**	58.62 ± 1.77 *^#^	2.34 ±0.43	15.26 ± 1.32	23.78 ± 1.18 *^#^
***p*-value**	*p* < 0.001	*p* = 0.483	*p* = 0.315	*p* < 0.001
**F1–Control**	64.51 ± 1.67	3.13 ± 0.39	18.50 ± 1.78	13.86 ± 2.12
**F1–55 mg/kg b.w./day**	64.74 ± 1.67	3.05 ± 0.81	21.65 ± 2.03	10.56 ± 1.47
***p*-value**	*p* = 0.924	*p* = 0.933	*p* = 0.258	*p* = 0.218

Values are mean ± standard error of the mean (SEM). Mean percentages of normal sperm morphology and percentages of abnormalities in the head, midpiece, and tail. * Significant differences in comparison with the respective control group. # Significant differences in comparison with their 14 mg/kg b.w./day group.

**Table 6:** Absolute organ weights (g) of parental F0 male and female CD1 mice fed with different doses of PTSO (0, 14, 28, and 55 mg/kg b.w./day). Values are mean and SD for 20 mice/sex/group.

Absolute Organ Weight Data Summary of Parental F0 Mice											
Parameters		Male				Parameters		Female			
		0	14	28	55			0	14	28	55
		N = 20	N = 20	N = 20	N = 20			N = 20	N = 20	N = 20	N = 20
**B**	**MEAN**	0.494	0.495	0.501	0.493	**B**	**MEAN**	0.485	0.505	0.496	0.501
	**SD**	0.027	0.043	0.033	0.024		**SD**	0.078	0.025	0.023	0.028
**L**	**MEAN**	2.439	2.582	2.279	2.293	**L**	**MEAN**	2.288	2.230	2.383	2.277
	**SD**	0.264	1.016	0.716	0.546		**SD**	0.362	0.391	0.593	0.341
**LK**	**MEAN**	0.385	0.346	0.353	0.399	**LK**	**MEAN**	0.232	0.245	0.246	0.247
	**SD**	0.050	0.024	0.070	0.057		**SD**	0.024	0.063	0.043	0.027
**RK**	**MEAN**	0.393	0.419	0.446	0.402	**RK**	**MEAN**	0.239	0.231	0.249	0.247
	**SD**	0.065	0.054	0.070	0.052		**SD**	0.024	0.020	0.030	0.027
**S**	**MEAN**	0.115	0.111	0.105	0.118	**S**	**MEAN**	0.151	0.168	0.153	0.143
	**SD**	0.025	0.030	0.029	0.012		**SD**	0.031	0.038	0.051	0.023
**LA**	**MEAN**	0.007	0.006	0.006	0.006	**LA**	**MEAN**	0.008	0.007	0.008	0.008
	**SD**	0.002	0.002	0.002	0.002		**SD**	0.002	0.002	0.002	0.004
**RA**	**MEAN**	0.005	0.005	0.005	0.006	**RA**	**MEAN**	0.007	0.008	0.007	0.008
	**SD**	0.001	0.001	0.002	0.002		**SD**	0.002	0.002	0.003	0.003
**P**	**MEAN**	0.030	0.035	0.033	0.035						
	**SD**	0.009	0.010	0.010	0.013						
**SV**	**MEAN**	0.575	0.604	0.595	0.582						
	**SD**	0.162	0.122	0.130	0.118						

Adrenal gland (left): LA; adrenal gland (right): RA; brain: B; epididymis (left): LE; epididymis (right): RE; kidney (left): LK; kidney (right): RK; liver: L; prostate: P; seminal vesicle: SV; spleen: S.

**Table 7:** Absolute organ weight (g), relative organ weight/body weight (%), and relative organ weight/brain weight (%) of parental F1 male and female CD1 mice fed with different doses of PTSO (0, 14, 28, and 55 mg/kg b.w./day). Values are mean and SD for 20 mice/sex/group.

Absolute Organ Weight Data Summary of Parental F1 Mice											
Parameters		Male				Parameters		Female			
		0	14	28	55			0	14	28	55
		N = 20	N = 20	N = 20	N = 20			N = 20	N = 20	N = 20	N = 20
**B**	**MEAN**	0.491	0.476	0.489	0.500	**B**	**MEAN**	0.505	0.484	0.498	0.489
	**SD**	0.026	0.030	0.026	0.029		**SD**	0.028	0.030	0.028	0.026
**L**	**MEAN**	2.431	2.600	2.592	2.469	**L**	**MEAN**	2.271	2.106	2.243	2.366
	**SD**	0.340	0.531	0.456	0.478		**SD**	0.489	0.288	0.349	0.453
**LK**	**MEAN**	0.413	0.355	0.383	0.351	**LK**	**MEAN**	0.255	0.229	0.254	0.253
	**SD**	0.099	0.051	0.051	0.059		**SD**	0.062	0.023	0.035	0.035
**RK**	**MEAN**	0.386	0.359	0.397	0.357	**RK**	**MEAN**	0.238	0.228	0.251	0.253
	**SD**	0.061	0.062	0.065	0.044		**SD**	0.034	0.022	0.034	0.040
**S**	**MEAN**	0.116	0.125	0.119	0.131	**S**	**MEAN**	0.150	0.147	0.154	0.150
	**SD**	0.025	0.034	0.040	0.030		**SD**	0.041	0.050	0.054	0.042
**LA**	**MEAN**	0.008	0.006	0.008	0.009	**LA**	**MEAN**	0.008	0.007	0.009	0.008
	**SD**	0.005	0.003	0.004	0.005		**SD**	0.004	0.003	0.003	0.004
**RA**	**MEAN**	0.008	0.006	0.007	0.007	**RA**	**MEAN**	0.007	0.009	0.009	0.009
	**SD**	0.004	0.002	0.003	0.004		**SD**	0.002	0.006	0.003	0.004
**LT**	**MEAN**	0.134	0.136	0.142	0.141	**U**	**MEAN**	0.233	0.197	0.194	0.208
	**SD**	0.020	0.035	0.028	0.017		**SD**	0.096	0.067	0.054	0.069
**RT**	**MEAN**	0.132	0.139	0.147	0.140	**LO**	**MEAN**	0.024	0.032	0.023	0.025
	**SD**	0.024	0.031	0.022	0.017		**SD**	0.007	0.016	0.005	0.009
**LE**	**MEAN**	0.063	0.060	0.074	0.077	**RO**	**MEAN**	0.022	0.026	0.028	0.028
	**SD**	0.016	0.019	0.032	0.029		**SD**	0.007	0.013	0.015	0.010
**RE**	**MEAN**	0.061	0.062	0.074	0.067						
	**SD**	0.014	0.019	0.030	0.024						
**P**	**MEAN**	0.050	0.053	0.052	0.051						
	**SD**	0.023	0.021	0.020	0.023						
**SV**	**MEAN**	0.473	0.516	0.527	0.476						
	**SD**	0.074	0.100	0.095	0.099						

Adrenal gland (left): LA; adrenal gland (right): RA; brain: B; epididymis (left): LE; epididymis (right): RE; kidney (left): LK; kidney (right): RK; liver: L; ovary (left): LO; ovary (right): RO; prostate: P; seminal vesicle: SV; spleen: S; testicle (left): LT; testicle (right): RT; uterus: U.

**Table 8:** Absolute organ weight (g), relative organ weight/body weight (%), and relative organ weight/brain weight (%) of F1 and F2 offspring male and female CD1 mice fed with different doses of PTSO (0, 14, 28, and 55 mg/kg b.w./day). Values are mean and SD for 20 mice/sex/group.

Absolute Organ Weight Data Summary of Offspring F1 Mice											
Parameters		Male				Parameters		Female			
		0	14	28	55			0	14	28	55
		N = 22	N = 20	N = 22	N = 22			N = 22	N = 21	N = 21	N = 22
**BW**	**MEAN**	19.020	18.422	18.849	18.215	**BW**	**MEAN**	15.880	16.141	15.136	15.127
	**SD**	4.663	4.314	3.364	3.524		**SD**	4.099	3.953	3.360	3.032
**B**	**MEAN**	0.434	0.435	0.437	0.426	**B**	**MEAN**	0.423	0.416	0.417	0.418
	**SD**	0.026	0.027	0.032	0.036		**SD**	0.044	0.042	0.040	0.032
**S**	**MEAN**	0.118	0.117	0.119	0.123	**S**	**MEAN**	0.101	0.105	0.104	0.104
	**SD**	0.044	0.033	0.030	0.026		**SD**	0.039	0.032	0.030	0.032
**T**	**MEAN**	0.100	0.093	0.087	0.086	**T**	**MEAN**	0.092	0.090	0.089	0.079
	**SD**	0.032	0.025	0.023	0.030		**SD**	0.029	0.028	0.021	0.029
**Absolute Organ Weight Data Summary of Offspring F2 Mice**											
**Parameters**		**Male**				**Parameters**		**Female**			
		**0**	**14**	**28**	**55**			**0**	**14**	**28**	**55**
		**N = 20**	**N = 20**	**N = 22**	**N = 21**			**N = 21**	**N = 20**	**N = 22**	**N = 21**
**BW**	**MEAN**	30.289	30.547	28.221	27.178	**BW**	**MEAN**	25.771	25.779	24.113	24.206
	**SD**	3.084	2.338	5.081	2.397		**SD**	2.494	3.11	2.154	2.483
**B**	**MEAN**	0.435	0.442	0.440	0.430	**B**	**MEAN**	0.435	0.411	0.442	0.416
	**SD**	0.037	0.020	0.035	0.020		**SD**	0.033	0.051	0.030	0.016
**S**	**MEAN**	0.110	0.114	0.113	0.110	**S**	**MEAN**	0.139	0.140	0.133	0.119
	**SD**	0.009	0.023	0.016	0.011		**SD**	0.031	0.041	0.030	0.020
**T**	**MEAN**	0.084	0.069	0.089	0.068	**T**	**MEAN**	0.091	0.080	0.094	0.084
	**SD**	0.081	0.015	0.025	0.018		**SD**	0.013	0.019	0.029	0.021

Brain: B; body weight: BW; spleen: S; thymus: T.

## Data Availability

Data is contained within the article and Supplementary Materials.

## Supplementary Materials

The following supporting information can be downloaded at https://www.mdpi.com/article/10.3390/antiox13030350/s1, Figure S1: Intake of PTSO of CD1 male and female mice fed with different doses of PTSO in the diet for two generations. Values are mean ± SD for 20 mice/group/sex. (A) Male F0, (B) Female F0, (C) Male F1, (D) Female F1. Table S1: Relative organ weight/body weights (%), and relative organ weight/brain weights (%) of parental F0 male and female CD1 mice fed with different doses of PTSO (0, 14, 28, and 55 mg/kg b.w./day). Values are mean and SD for 20 mice/sex/group. Table S2: Relative organ weight/body weights (%), and relative organ weight/brain weights (%) of parental F1 male and female CD1 mice fed with different doses of PTSO (0, 14, 28, and 55 mg/kg b.w./day). Values are mean and SD for 20 mice/sex/group. Table S3: Relative organ weight/body weights (%), and relative organ weight/brain weights (%) of F1 and F2 offspring male and female CD1 mice fed with different doses of PTSO (0, 14, 28, and 55 mg/kg b.w./day). Values are mean and SD for 20 mice/sex/group. The coded symbols represent the following: #—statistical differences compared with 14 mg PTSO/kg b. w./day.
