# Supplementary material for: Two-Generation Toxicity Study of the Antioxidant Compound Propyl-Propane Thiosulfonate (PTSO)
Source: Antioxidants (Basel). 2024 Mar 15;13(3):350. doi: 10.3390/antiox13030350 (PMC10968083; doi:10.3390/antiox13030350)
Supplement: Supplementary file 1 [file antioxidants-13-00350-s001.zip › Tables S1-S3.pdf]

Table S1. Relative organ weight/body weights (%), and relative organ weight/brain weights (%) of parental F0 male and female CD1 mice fed with different doses of PTSO (0, 14, 28 and 55 mg/kg b.w./day). Values are mean and SD for 20 mice/sex/group.

**ORGAN WEIGHT TO B.W. RATIOS DATA SUMMARY OF PARENTAL F0 MICE**											
**PARAMETERS**		MALE				**PARAMETERS**		FEMALE			
		**0**	**14**	**28**	**55**			**0**	**14**	**28**	**55**
		**N=20**	**N=20**	**N=20**	**N=20**			**N=20**	**N=20**	**N=20**	**N=20**
**B**	**MEAN**	1.030	1.086	1.053	0.996	**B**	**MEAN**	1.227	1.257	1.211	1.269
	**SD**	0.095	0.178	0.167	0.140		**SD**	0.216	0.148	0.148	0.121
**L**	**MEAN**	5.028	5.511	4.938	4.553	**L**	**MEAN**	5.742	5.469	5.719	5.669
	**SD**	0.367	1.707	0.880	0.948		**SD**	0.680	0.637	1.119	0.640
**LK**	**MEAN**	0.802	0.700	0.798	0.796	**LK**	**MEAN**	0.584	0.607	0.595	0.620
	**SD**	0.112	0.192	0.147	0.097		**SD**	0.054	0.161	0.100	0.055
**RK**	**MEAN**	0.822	0.866	0.992	0.804	**RK**	**MEAN**	0.602	0.572	0.609	0.614
	**SD**	0.147	0.091	0.159	0.089		**SD**	0.051	0.048	0.079	0.040
**S**	**MEAN**	0.219	0.238	0.231	0.220	**S**	**MEAN**	0.381	0.411	0.368	0.357
	**SD**	0.072	0.050	0.059	0.092		**SD**	0.070	0.074	0.113	0.065
**LA**	**MEAN**	0.014	0.011	0.012	0.013	**LA**	**MEAN**	0.019	0.018	0.020	0.021
	**SD**	0.004	0.006	0.004	0.004		**SD**	0.004	0.005	0.004	0.008
**RA**	**MEAN**	0.012	0.010	0.011	0.011	**RA**	**MEAN**	0.019	0.020	0.017	0.021
	**SD**	0.003	0.002	0.004	0.006		**SD**	0.004	0.006	0.006	0.007
**P**	**MEAN**	0.062	0.075	0.075	0.069						
	**SD**	0.018	0.017	0.020	0.025						
**SV**	**MEAN**	1.213	1.311	1.306	1.166						
	**SD**	0.423	0.245	0.250	0.237						
**ORGAN WEIGHT TO BRAIN RATIOS DATA SUMMARY OF PARENTAL F0 MICE**											
**PARAMETERS**		MALE				**PARAMETERS**		FEMALE			
		**0**	**14**	**28**	**55**			**0**	**14**	**28**	**55**
		**N=20**	**N=20**	**N=20**	**N=20**			**N=20**	**N=20**	**N=20**	**N=20**
**L**	**MEAN**	491.909	526.185	458.240	464.944	**B**	**MEAN**	498.184	441.984	482.873	455.115
	**SD**	56.351	219.669	153.279	111.834		**SD**	189.359	90.869	127.586	68.717
**LK**	**MEAN**	78.093	69.467	70.926	80.984	**L**	**MEAN**	50.486	48.685	49.764	49.366
	**SD**	9.422	7.265	15.319	12.435		**SD**	19.048	14.214	9.672	5.495
**RK**	**MEAN**	79.870	85.395	89.643	81.713	**LK**	**MEAN**	51.446	45.774	50.278	49.421
	**SD**	12.928	9.466	16.991	11.839		**SD**	15.452	5.389	7.352	5.721
**S**	**MEAN**	21.460	22.444	20.879	24.280	**RK**	**MEAN**	32.630	33.745	27.833	28.731
	**SD**	6.973	6.218	5.933	2.239		**SD**	11.420	8.536	14.039	5.022
**LA**	**MEAN**	1.349	1.071	1.107	1.279	**S**	**MEAN**	1.626	1.382	1.631	1.655
	**SD**	0.405	0.467	0.419	0.393		**SD**	0.739	0.399	0.329	0.695
**RA**	**MEAN**	1.104	0.956	0.960	1.215	**LA**	**MEAN**	1.682	1.585	1.431	1.663
	**SD**	0.301	0.194	0.319	0.354		**SD**	0.961	0.480	0.506	0.594
**P**	**MEAN**	6.006	7.069	6.637	7.142						
	**SD**	1.808	2.068	2.021	2.842						
**SV**	**MEAN**	118.457	123.194	118.682	118.411						
	**SD**	38.622	27.001	25.130	25.023						

Adrenal Gland (Left): LA; Adrenal Gland (Right): RA; Brain: B; Epididymis (Left): LE; Epididymis (Right): RE; Kidney (Left): LK; Kidney (Right): RK; Liver: L; Prostate: P; Seminal Vesicle: SV; Spleen: S.

Table S2. Relative organ weight/body weights (%), and relative organ weight/brain weights (%) of parental F1 male and female CD1 mice fed with different doses of PTSO (0, 14, 28 and 55 mg/kg b.w./day). Values are mean and SD for 20 mice/sex/group.

**ORGAN WEIGHT TO B.W. RATIO DATA SUMMARY OF PARENTAL F1 MICE**											
**PARAMETERS**		MALE				**PARAMETERS**		FEMALE			
		**0**	**14**	**28**	**55**			**0**	**14**	**28**	**55**
		**N=20**	**N=20**	**N=20**	**N=20**			**N=20**	**N=20**	**N=20**	**N=20**
**B**	**MEAN**	1.068	0.964	0.957	1.057	**B**	**MEAN**	1.289	1.207	1.236	1.186
	**SD**	0.119	0.185	0.081	0.161		**SD**	0.198	0.149	0.173	0.148
**L**	**MEAN**	5.243	5.119	4.967	5.102	**L**	**MEAN**	5.675	5.194	5.515	5.679
	**SD**	0.528	0.599	0.668	0.404		**SD**	0.784	0.480	0.747	0.951
**LK**	**MEAN**	0.816	0.722	0.735	0.754	**LK**	**MEAN**	0.643	0.568	0.627	0.611
	**SD**	0.076	0.117	0.078	0.132		**SD**	0.149	0.069	0.097	0.099
**RK**	**MEAN**	0.832	0.754	0.761	0.737	**RK**	**MEAN**	0.599	0.569	0.622	0.614
	**SD**	0.087	0.126	0.093	0.133		**SD**	0.072	0.054	0.098	0.114
**S**	**MEAN**	0.252	0.247	0.226	0.278	**S**	**MEAN**	0.377	0.368	0.376	0.361
	**SD**	0.049	0.051	0.066	0.090		**SD**	0.092	0.143	0.120	0.106
**LA**	**MEAN**	0.016	0.011	0.015	0.017	**LA**	**MEAN**	0.020	0.017	0.022	0.020
	**SD**	0.011	0.006	0.007	0.010		**SD**	0.008	0.008	0.007	0.010
**RA**	**MEAN**	0.018	0.013	0.014	0.016	**RA**	**MEAN**	0.019	0.024	0.023	0.021
	**SD**	0.009	0.004	0.005	0.008		**SD**	0.005	0.014	0.007	0.011
**LT**	**MEAN**	0.292	0.271	0.274	0.299	**U**	**MEAN**	0.595	0.486	0.487	0.499
	**SD**	0.051	0.068	0.060	0.063		**SD**	0.262	0.164	0.163	0.160
**RT**	**MEAN**	0.286	0.278	0.271	0.295	**LO**	**MEAN**	0.061	0.066	0.056	0.062
	**SD**	0.056	0.070	0.082	0.047		**SD**	0.015	0.032	0.013	0.023
**LE**	**MEAN**	0.131	0.116	0.143	0.164	**RO**	**MEAN**	0.056	0.080	0.066	0.067
	**SD**	0.051	0.027	0.066	0.064		**SD**	0.020	0.043	0.028	0.024
**RE**	**MEAN**	0.125	0.123	0.143	0.142						
	**SD**	0.044	0.034	0.060	0.051						
**P**	**MEAN**	0.106	0.104	0.095	0.107						
	**SD**	0.047	0.035	0.046	0.046						
**SV**	**MEAN**	1.017	1.028	1.017	1.002						
	**SD**	0.094	0.186	0.182	0.214						

**ORGAN WEIGHT TO BRAIN RATIO DATA SUMMARY OF PARENTAL F1 MICE**											
**PARAMETERS**		MALE				**PARAMETERS**		FEMALE			
		**0**	**14**	**28**	**55**			**0**	**14**	**28**	**55**
		**N=20**	**N=20**	**N=20**	**N=20**			**N=20**	**N=20**	**N=20**	**N=20**
**L**	**MEAN**	496.107	547.793	530.050	493.302	**L**	**MEAN**	452.506	435.046	451.618	485.054
	**SD**	73.530	118.418	88.028	84.901		**SD**	108.454	54.441	73.560	98.720
**LK**	**MEAN**	83.978	74.683	78.282	71.643	**LK**	**MEAN**	50.579	47.267	51.056	51.855
	**SD**	18.056	11.471	9.857	9.213		**SD**	12.339	4.688	7.276	8.259
**RK**	**MEAN**	78.643	75.598	78.844	70.274	**RK**	**MEAN**	47.238	47.326	50.545	51.913
	**SD**	10.938	14.325	10.278	11.475		**SD**	7.824	3.897	7.012	8.949
**S**	**MEAN**	23.673	26.419	24.356	26.087	**S**	**MEAN**	29.619	30.339	30.964	30.891
	**SD**	4.602	7.585	7.936	5.540		**SD**	7.948	9.881	11.454	9.172
**LA**	**MEAN**	1.538	1.120	1.574	1.567	**LA**	**MEAN**	1.593	1.457	1.815	1.671
	**SD**	1.080	0.678	0.790	0.946		**SD**	0.800	0.730	0.687	0.947
**RA**	**MEAN**	1.715	1.308	1.513	1.529	**RA**	**MEAN**	1.468	1.987	1.897	1.813
	**SD**	0.899	0.678	0.494	0.701		**SD**	0.464	1.262	0.659	0.967
**LT**	**MEAN**	27.509	28.688	28.917	28.278	**U**	**MEAN**	45.850	40.875	38.697	42.501
	**SD**	4.632	7.594	5.224	3.914		**SD**	17.786	13.352	9.572	14.505
**RT**	**MEAN**	26.987	29.158	28.586	28.121	**LO**	**MEAN**	4.813	5.387	4.563	5.177
	**SD**	5.365	6.757	7.830	3.602		**SD**	1.472	2.481	0.992	1.862
**LE**	**MEAN**	12.174	12.621	15.032	15.477	**RO**	**MEAN**	4.355	6.629	5.508	5.681

	**SD**	4.269	4.339	6.309	5.784		**SD**	1.336	3.413	2.834	2.018
**RE**	**MEAN**	11.691	12.998	15.084	13.413						
	**SD**	3.955	4.104	5.858	4.583						
**P**	**MEAN**	10.162	11.248	10.164	10.145						
	**SD**	4.734	4.718	4.826	4.431						
**SV**	**MEAN**	96.257	108.891	108.222	95.672						
	**SD**	13.750	24.104	20.890	20.479						

Adrenal Gland (Left): LA; Adrenal Gland (Right): RA; Brain: B; Epididymis (Left): LE; Epididymis (Right): RE; Kidney (Left): LK; Kidney (Right): RK; Liver: L; Ovary (Left): LO; Ovary (Right): RO; Prostate: P; Seminal Vesicle: SV; Spleen: S; Testicle (Left): LT; Testicle (Right): RT; Uterus: U.

Table S3. Relative organ weight/body weights (%), and relative organ weight/brain weights (%) of F1 and F2 offspring male and female CD1 mice fed with different doses of PTSO (0, 14, 28 and 55 mg/kg b.w./day). Values are mean and SD for 20 mice/sex/group. The coded symbols represent the following: #—statistical differences compared with 14 mg PTSO/kg b. w./day.

**ORGAN WEIGHT TO B.W. RATIO DATA SUMMARY OF OFFSPRING F1 MICE**											
**PARAMETERS**		MALE						FEMALE			
		**0**	**14**	**28**	**55**	**PARAMETERS**		**0**	**14**	**28**	**55**
		**N=22**	**N=20**	**N=22**	**N=22**			**N=22**	**N=21**	**N=21**	**N=22**
**B**	**MEAN**	2.379	2.473	2.394	2.463	**B**	**MEAN**	2.798	2.737	2.857	2.847
	**SD**	0.439	0.518	0.459	0.442		**SD**	0.580	0.792	0.514	0.487
**S**	**MEAN**	0.636	0.653	0.643	0.772	**S**	**MEAN**	0.621	0.671	0.689	0.711
	**SD**	0.220	0.162	0.143	0.182		**SD**	0.186	0.189	0.191	0.235
**T**	**MEAN**	0.541	0.510	0.447	0.573	**T**	**MEAN**	0.583	0.558	0.600	0.541
	**SD**	0.152	0.119	0.103	0.117		**SD**	0.155	0.155	0.136	0.211
**ORGAN WEIGHT TO BRAIN RATIO DATA SUMMARY OF OFFSPRING F1 MICE**											
**PARAMETERS**		PARAMETERS						PARAMETERS			
		**0**	**14**	**28**	**55**	**PARAMETERS**		**0**	**14**	**28**	**55**
		**N=22**	**N=20**	**N=22**	**N=22**			**N=22**	**N=21**	**N=21**	**N=22**
**S**	**MEAN**	27.129	26.671	27.293	29.048	**S**	**MEAN**	23.364	25.187	24.694	24.746
	**SD**	9.611	6.364	6.441	6.500		**SD**	8.154	7.014	6.349	7.031
**T**	**MEAN**	26.371	21.387	19.860	20.577	**T**	**MEAN**	21.432	21.386	21.191	18.790
	**SD**	7.029	5.673	5.319	6.526		**SD**	5.964	6.432	4.332	6.476
**ORGAN WEIGHT TO B.W. RATIO DATA SUMMARY OF OFFSPRING F2 MICE**											
**PARAMETERS**		MALE						FEMALE			
		**0**	**14**	**28**	**55**	**PARAMETERS**		**0**	**14**	**28**	**55**
		**N=20**	**N=20**	**N=22**	**N=21**			**N=21**	**N=20**	**N=22**	**N=21**
**B**	**MEAN**	1.442	1.430	1.617	1.570#	**B**	**MEAN**	1.647	1.600	1.766#	1.733#
	**SD**	0.098	0.091	0.364	0.077		**SD**	0.136	0.134	0.116	0.116
**S**	**MEAN**	0.359	0.373	0.419	0.432	**S**	**MEAN**	0.562	0.541	0.562	0.497
	**SD**	0.055	0.071	0.074	0.017		**SD**	0.111	0.137	0.118	0.100
**T**	**MEAN**	0.227	0.227	0.297	0.274	**T**	**MEAN**	0.344	0.312	0.405	0.354
	**SD**	0.248	0.053	0.084	0.029		**SD**	0.054	0.090	0.116	0.103
**ORGAN WEIGHT TO BRAIN RATIO DATA SUMMARY OF OFFSPRING F2 MICE**											
**PARAMETERS**		MALE						FEMALE			
		**0**	**14**	**28**	**55**	**PARAMETERS**		**0**	**14**	**28**	**55**
		**N=20**	**N=20**	**N=22**	**N=21**			**N=21**	**N=20**	**N=22**	**N=21**
**S**	**MEAN**	25.508	25.386	25.931	27.139	**S**	**MEAN**	32.745	34.402	31.585	28.611
	**SD**	8.949	5.168	2.387	2.572		**SD**	6.252	10.456	7.210	4.725
**T**	**MEAN**	19.051	15.119	21.301	16.278	**T**	**MEAN**	20.928	19.896	22.329	20.304
	**SD**	16.441	2.796	5.961	4.359		**SD**	2.710	6.399	7.159	5.093

Brain: B; Spleen: S; Thymus: T.
