# Supplementary figures and images for: Two-Generation Toxicity Study of the Antioxidant Compound Propyl-Propane Thiosulfonate (PTSO)
Source: Antioxidants (Basel). 2024 Mar 15;13(3):350. doi: 10.3390/antiox13030350 (PMC10968083; doi:10.3390/antiox13030350)

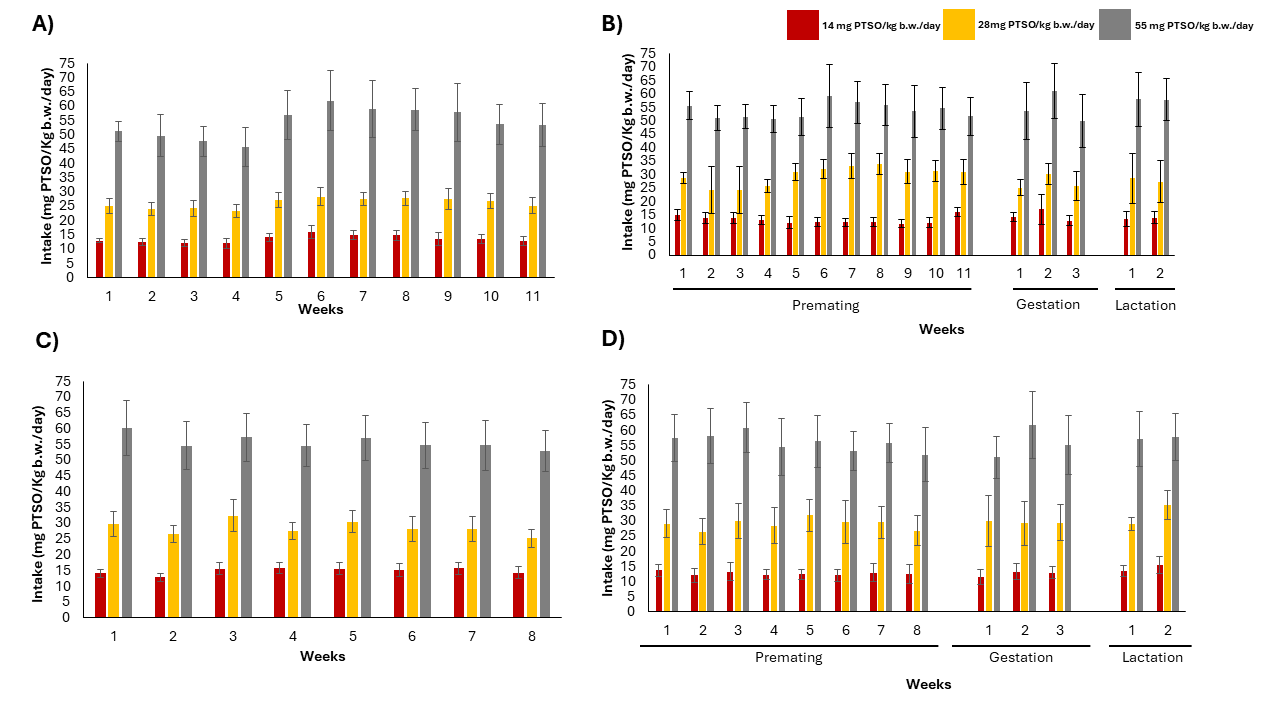

Supplement: Supplementary file 1 [file antioxidants-13-00350-s001.zip › Figure S1.tif]
